# Quantifying local pH changes in carbonate electrolyte during copper-catalysed $\hbox {CO}_2$ electroreduction using *in operando* $^{13}\hbox {C}$ NMR

**DOI:** 10.1038/s41598-022-12264-8

**Published:** 2022-05-18

**Authors:** Michael Schatz, Sven Jovanovic, Rüdiger-A. Eichel, Josef Granwehr

**Affiliations:** 1 Institute of Energy and Climate Research, Fundamental Electrochemistry (IEK-9), Forschungszentrum Jülich, 52425 Jülich, Germany; 2 Institute of Technical and Macromolecular Chemistry, RWTH Aachen University, 52074 Aachen, Germany; 3 Institute of Physical Chemistry, RWTH Aachen University, 52074 Aachen, Germany

**Keywords:** Chemical physics, Solution-state NMR, Electrocatalysis

## Abstract

The electrochemical carbon dioxide reduction on copper attracted considerable attention within the last decade, since Cu is the only elemental transition metal that catalyses the formation of short-chain hydrocarbons and alcohols. Research in this field is mainly focused on understanding the reaction mechanism in terms of adsorbates and intermediates. Furthermore, dynamic changes in the micro-environment of the catalyst, i.e. local pH and $\hbox {CO}_2$ concentration values, play an equivalently important role in the selectivity of product formation. In this study, we present an *in operando* $^{13}\hbox {C}$ nuclear magnetic resonance technique that enables the simultaneous measurement of pH and $\hbox {CO}_2$ concentration in electrode vicinity during electroreduction. The influence of applied potential and buffer capacity of the electrolyte on the formation of formate is demonstrated. Theoretical considerations are confirmed experimentally and the importance of the interplay between catalyst and electrolyte is emphasised.

## Introduction

In recent years, the electrochemical $\hbox {CO}_2$ reduction reaction ($\hbox {CO}_2$RR) has been recognised as a possible industrially applicable contribution for establishing a closed carbon cycle^1^. Driven by intermittent renewable electricity, this process has the potential to produce carbon-neutral fuels and feedstock chemicals while simultaneously stabilising the electric grid by acting as energy store^2^.

In addition to electrode surface characteristics and reaction conditions, the most influential factors for $\hbox {CO}_2$RR are properties of the aqueous solution surrounding the electrode, i.e. local pH and $\hbox {HCO}_3^-$/$\hbox {CO}_2$ concentration values^3–5^. How these local properties are affected by applied potential and how this in turn influences product formation has not been addressed experimentally owing to a lack of suitable *in operando* methods^6^.

Among metal catalysts for $\hbox {CO}_2$RR, elemental copper has the unique ability to catalyse short-chain hydrocarbon evolution. In order to optimise future copper-based electrocatalyst designs, the understanding of underlying reaction mechanisms needs to be improved^7,8^. The first publication about Cu-catalysed $\hbox {CO}_2$RR by Hori et al.^7^ discussed the interdependence of hydrocarbon and alcohol formation on local changes of the $\hbox {KHCO}_3$ buffer. In dilute $\hbox {KHCO}_3$ solution, the pH in cathode proximity increases due to $\hbox {OH}^-$ formation as part of the reduction reaction as well as poor buffer capacity of the solution. This in turn prevents the hydrogen evolution reaction (HER) and promotes the reduction of $\hbox {CO}_2$. If a potential more negative than $-1.1\,{\mathrm {V}}$ versus normal hydrogen electrode is applied, CO stays adsorbed at the cathode and can be further reduced to C_2+_ products, e.g. ethylene, ethanol or even n-propanol^7^. Gupta et al. presented calculations investigating the interplay between local pH, buffer capacity and current density. For a bulk pH of 6.81 and a current density of 10 A/m$^2$, they determined a pH of up to 9 on the electrode surface. Due to the shifted $\hbox {CO}_2$/$\hbox {HCO}_3^-$ equilibrium and simultaneous reduction of $\hbox {CO}_2$ in electrode proximity, a decrease of the local $\hbox {CO}_2$ concentration by a factor of 2.1 was predicted^9^. However, a decrease in ${\hbox {CO}_2}$ concentration does not translate linearly to a decrease in conversion rate. It has been shown using $^{13}\hbox {C}$ labelling that the main source of converted carbon is $\hbox {CO}_2$ from the equilibrium with $\hbox {HCO}_3^-$^10^. This led to the conclusion that the effective concentration of $\hbox {CO}_2$ in electrode vicinity is equivalent to the bulk^11^. Nonetheless, local $\hbox {CO}_2$ scarcity and basic pH dictate the reaction conditions at the electrode and must be considered for understanding the reaction mechanism of $\hbox {CO}_2$RR on copper.

Local pH effects have proven important in shifting the selectivity to desired products. A high local pH can be advantageous for reaction pathways that include a rate determining step without a proton transfer. In this case, hydrogen formation is hindered while product formation is pH-independent. This applies e.g. to the C–C formation step in the reaction pathway to ethylene and ethanol^12–14^. In contrast, the formation of products such as methane that include a proton transfer in the rate determining step are inhibited^5^. Local shifts in the concentrations of $\hbox {CO}_2$ and $\hbox {HCO}_3^-$ also affect the product formation. At small negative overpotential and therefore only modest changes in local pH, formate is formed in the presence of adsorbed $\hbox {CO}_3^{2-}$. More negative potentials shift the equilibrium to solution-based bicarbonate and promote CO formation^15,16^.

The effects of local conditions have been utilised in electrode engineering. Roughened or porous electrode surfaces promote high local pH, which can effectively suppress HER^14^ and simultaneously favour $\hbox {C}_{2+}$ product formation by confinement of intermediate products^5,13^. However, a study using a Cu nanofoam electrode showed increased formate production over the whole potential range, suppressing the pathways to methane and ethylene almost completely^17^. This contradiction shows how important it is to understand the complex interplay between electrolyte composition, applied potential and surface morphology for electrode engineering. In order to generate a micro-environment that is suitable for the desired product formation, all of these parameters have to be considered^18^. Burdyny et al. even suggest considering the catalyst as combined surface and electrolyte system rather than just a metal surface^19^. To understand the catalytic performance of such a surface/electrolyte system, it is indispensable to know how local pH evolves during operation, and how the $\hbox {CO}_2$ concentration and therefore the accessibility of the reactant varies locally.

Measuring local concentrations of protons and molecules that take part in electrochemical reactions has been attempted, for example, using scanning probe techniques and various optical methods^20^. Recent studies investigating $\hbox {CO}_2$RR apply scanning electrochemical microscopy (SECM)^21^, surface-enhanced infrared absorption spectroscopy (SEIRAS)^22,23^ and surface-enhanced Raman spectroscopy (SERS)^24^. SECM offers direct measurement of the proton concentration by using the pH-sensitive electrochemical response of an inert metallic material. The SEIRAS and SERS methods measure the pH by monitoring the ratio of the species composing a buffer system and are, therefore, indirect measurement methods^20^. In these experiments, typical buffer solutions for $\hbox {CO}_2$RR such as $\hbox {CO}_2$(aq)/$\hbox {HCO}_3^-$/$\hbox {CO}_3^{2-}$^21,22,24^ and $\hbox {H}_2\hbox {PO}_4^-$/$\hbox {HPO}_4^{2-}$/$\hbox {PO}_4^{3-}$^23^ were investigated.

In this study, the measurement of pH is demonstrated in an electrochemical cell and during $\hbox {CO}_2$ electrolysis by *in operando* $^{13}\hbox {C}$ nuclear magnetic resonance (NMR), which has proven capable of investigating the $\hbox {CO}_2$(aq)/$\hbox {HCO}_3^-$/$\hbox {CO}_3^{2-}$ equilibrium in such an environment^25^. The method takes advantage of the high spectral resolution and sensitivity afforded by this cell design even in the presence of electrically conductive material^26^. In contrast to methods that only consider either the $\hbox {CO}_2$(aq)/$\hbox {HCO}_3^-$^22^ or the $\hbox {HCO}_3^-$/$\hbox {CO}_3^{2-}$ equilibrium^24^, the presented method provides data for a wider pH range by considering both equilibria, including an overlap region in-between. The sensitive volume of *in operando* NMR measurements is not limited to a few nanometres from the electrode as for surface-enhanced optical methods, and spatial resolution can be obtained by applying magnetic resonance imaging techniques. Furthermore, NMR spectroscopy can provide a variety of additional information, e.g. about sample chemistry, mobility and structure.

To calculate the pH from *in operando* $^{13}\hbox {C}$ NMR data, we utilise the buffer capability of $\hbox {HCO}_3^-$/$\hbox {CO}_3^{2-}$. Depending on the pH, either the equilibrium between $\hbox {HCO}_3^-$ and $\hbox {H}_2\hbox {CO}_3$, or the equilibrium between $\hbox {HCO}_3^-$ and $\hbox {CO}_3^{2-}$ is dominant^3^,

1 $$\begin{aligned}&{\hbox {CO}_3}^{2-} + \hbox {H}^+ \rightleftharpoons \hbox {HCO}_3^- \, , \quad {\mathrm {p}}K_{a1} = 10.3 \, , \end{aligned}$$

2 $$\begin{aligned}&\hbox {HCO}_3^- + \hbox {H}^+ \rightleftharpoons {\hbox {H}_2\hbox {CO}_3} \quad \Big ( \rightleftharpoons \hbox {CO}_2{{(\mathrm{{aq}})}} + \hbox {H}_2\hbox {O}\Big ) \, , \quad {\mathrm {p}}K_{a2} = 6.4 \, . \end{aligned}$$

Moret et al. have shown that due to the fast exchange between $\hbox {HCO}_3^-$ and $\hbox {CO}_3^{2-}$ compared to the NMR timescale given by the chemical shift difference, the $^{13}\hbox {C}$ resonances coalesce into a single peak with a pH dependent chemical shift. Hence, the term ‘carbonate’ is used in the following to describe both $\hbox {HCO}_3^-$ and $\hbox {CO}_3^{2-}$ in solution. The respective molecular formula is used when referring to a specific species. Alternatively, Scholz et al. estimated the pH in near neutral conditions using the Hendersson–Hasselbalch (HH) equation^27^,

3 $$\begin{aligned} {\mathrm {pH}} = {\mathrm {p}}K_{a2} + \log _{10} \frac{[\hbox {HCO}_3^-]}{[\hbox {CO}_2]} \, . \end{aligned}$$

Here, both methods are combined to assess the possibility of pH measurements over a wide range from neutral to basic environments. This technique is then applied *in operando* by varying potential and electrolyte concentration for a comparison with theoretical predictions, and to obtain new insight about the interplay between local pH, $\hbox {CO}_2$ accessibility, and buffer capacity.

## Results and discussion

**Figure 1:**
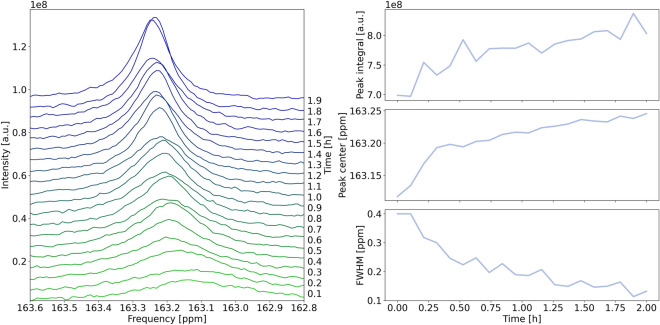
$^{13}\hbox {C}$ NMR resonance of carbonate during electrolysis at $-1.47$ V, with an initial $\hbox {KHCO}_3$ concentration of 0.1 M. (**a**) Evolution of the carbonate peak during 2 h of electrolysis. (**b**) Temporal evolution of the integral (top), $^{13}\hbox {C}$ chemical shift (middle), and FWHM (bottom) of the Lorentzian function fitted to the carbonate peak. Further data representing experiments at different potentials and concentrations are shown in Figs. S3 and S4.

In the $^{13}\hbox {C}$ NMR spectra, the three observed singlet resonances are assigned to the $^{13}\hbox {C}$-labelled methyl carbon of the acetonitrile reference, to $\hbox {CO}_2$(aq), and to a coalesced resonance of $\hbox {HCO}_3^-$ and $\hbox {CO}_3^{2-}$ due to fast exchange between these species. The peak properties of the three $^{13}\hbox {C}$ signals, i.e. integral, position (chemical shift) and full width at half maximum (FWHM), were determined by peak fitting using a Lorentzian function, which provided an adequate fit. For error estimation, fluctuations of the fitted values are determined in terms of standard deviation using a 5-step moving average. Details about the error estimation are available in Sect. S3 and Fig. S6 in the Supporting Information. Figure 1a shows the $^{13}\hbox {C}$ resonance of carbonate at $-1.47\,{\mathrm {V}}$ as a function of time, with an initial $\hbox {KHCO}_3$ concentration of 0.1 M. The evolution of the fitted peak properties is shown in Fig. 1b. The carbonate chemical shift (CCS) moves downfield from an initial value of 163.12 ppm, while the integral of the carbonate peak increases. This constitutes a shift of the $\hbox {HCO}_3^-$/$\hbox {CO}_2$ equilibrium towards $\hbox {HCO}_3^-$, caused by an increasing local pH. The FWHM decreases due to faster exchange between carbonate species, which has been observed before by *in operando* NMR studies with silver working electrodes^25^. The fitting parameters exhibit fluctuations that can be attributed to gas bubble formation on the electrode during the electrochemical experiments. The magnetic susceptibility of gas bubbles differs markedly from the surrounding electrolyte, causing inhomogeneities in the magnetic field that are perceived as variations of the peak shape. Furthermore, the quality factor of the resonant circuit used for radio frequency excitation and detection changes, leading to an additional fluctuation of the integral. The fluctuations of the resonance line parameters are $0.0192\pm 0.006\,{\mathrm {ppm}}$ for CCS, and $0.0281\pm 0.007\,{\mathrm {ppm}}$ for FWHM. The fluctuations of the integral relative to its initial value is $3.58\pm 0.63\,\%$.

The integral of the $\hbox {CO}_2$ resonance at 127.5 ppm decreases, approaching zero during experiments with high negative potential. Relative to its initial value, the fluctuations of the integral are $7.80\pm 0.88\,\%$. Chemical shift and width stay constant. This is expected and confirms that the origin of the aforementioned carbonate resonance shift is a variation of the $\hbox {HCO}_3^-$/$\hbox {CO}_2$ equilibrium.

Acetonitrile as reference substance is isolated by a glass capillary and consequently does not interact with other species during electrolysis. Hence, chemical shift, integral and width of its $^{13}\hbox {C}$-labelled methyl group are unaffected by the electrolysis reaction itself. Still, the formation of gas bubbles leads to fluctuations of the line shape. Fluctuations of chemical shift and FWHM are $0.0177\pm 0.005\,{\mathrm {ppm}}$ and $0.035\pm 0.01\,{\mathrm {ppm}}$, respectively. If these fluctuations would be exclusively caused by temperature fluctuations of the sample, the former would correspond to a change in temperature of about $0.1\,^{\circ }\hbox {C}$.

**Figure 2:**
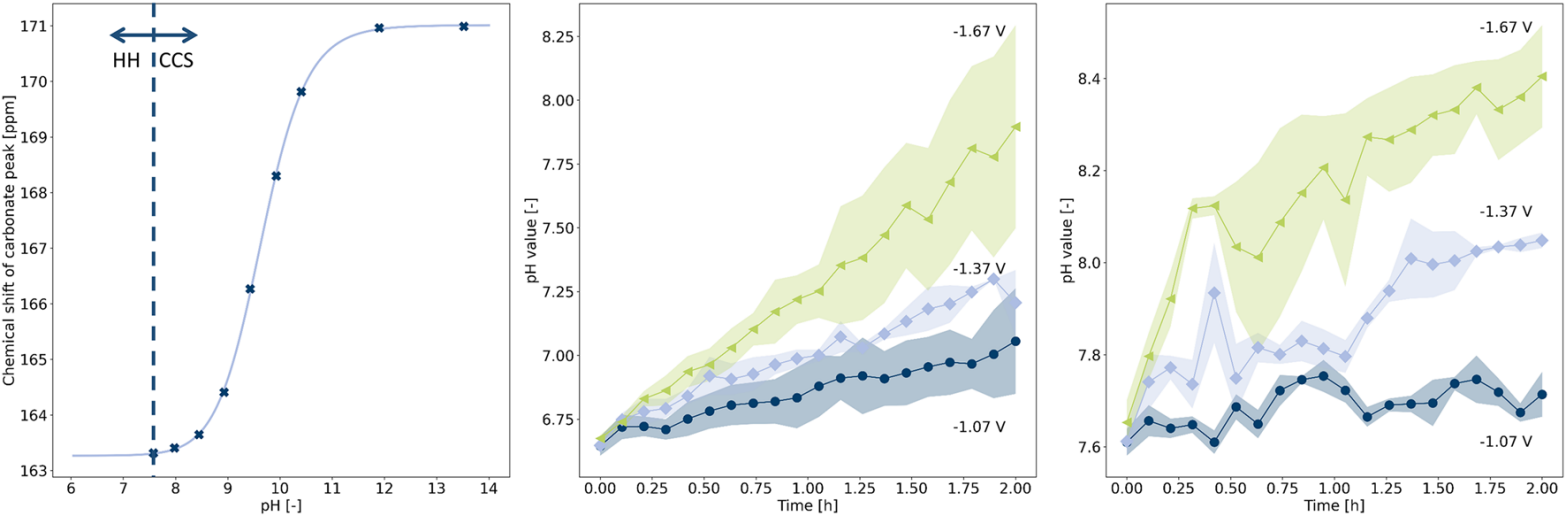
pH calculation using the CCS for ${\mathrm {pH}} \ge 7.6$ and the HH equation for ${\mathrm {pH}}< 7.6$. (**a**) pH dependent CCS at $10\,^{\circ }\hbox {C}$. Data points are marked by crosses and the fitted sigmoidal function by a solid line. (**b**, **c**) pH values as a function of time for initial electrolyte concentration of 0.1 M (**b**) and 1 M (**c**). Data points represent average value of experiments with an applied potential of $-1.07\,{\mathrm {V}}$ (dark blue), $-1.37\,{\mathrm {V}}$ (light blue) and $-1.67\,{\mathrm {V}}$ (green). Coloured areas show minimum and maximum value of the respective data point in two repetitions of the experiments. Further data representing experiments at other potentials are available in Fig. S5.

To calculate the local pH from the carbonate chemical shift $\delta _c$, a reference curve for the pH-dependence was recorded by titration of a 1 M $\hbox {KHCO}_3$ solution with 1 M KOH, which resulted in a typical sigmoidal curve depicted in Fig. 2a that was fitted by

4 $$\begin{aligned} \delta _c = \delta _\mathrm{{HCO}_3^-} + \frac{ \delta _{\mathrm{{CO}_3^{2-}}}-\delta _{\mathrm{{HCO}_3^-}} }{ 1 + 10^{\mathrm{{p}}K_{a1} - \mathrm{{pH}}} } \, . \end{aligned}$$

Here, $\delta _{{{\mathrm {HCO}}_3^-}}$ and $\delta _{{{\mathrm {CO}}_3^{2-}}}$ represent the chemical shifts of the pure respective species and are fitted as $\delta _{\mathrm {HCO}_3^-} = 163.267\pm 0.036\,\text {ppm}$, $\delta _{\mathrm {CO}_3^{2-}} = 171.011\pm 0.037\,\text {ppm}$. The fitted ${\mathrm {p}}K_{a1}$ value was $9.645\pm 0.011$. This function is derived from the carbonate equilibrium (see Sect. S1 of the Supporting Information). Equation 4 can be transformed to obtain an expression for the pH value,

5 $$\begin{aligned} {\mathrm {pH}} = {\mathrm {p}}K_{a1} + \log _{10} \left| \frac{\delta _c - \delta _{\mathrm {HCO}_3^-}}{\delta _c - \delta _{\mathrm {CO}_3^{2-}}}\right| \, . \end{aligned}$$

Using the titration curve, a lower threshold of ${\mathrm {pH}} = 7.6$ for reliable pH estimation by means of the CCS was obtained. Below this threshold, changes of the CCS are small compared to the measurement accuracy, and the equilibrium between $\hbox {CO}_2$(aq) and $\hbox {HCO}_3^-$ is dominant. Therefore, the HH Eq. (3) is more accurate for the determination of the pH value below ${\mathrm {pH}} = 7.6$. Here, the ratio of the integrals of $\hbox {CO}_2$(aq) and carbonate peak can be used equivalently to the ratio of the concentrations of the respective species^27^.

The values for ${\mathrm {p}}K_{a1}$ and ${\mathrm {p}}K_{a2}$ vary from literature data, since experiments are carried out at $10\,^{\circ }\hbox {C}$. Other influencing factors are the ionic strength of the electrolyte and the $\hbox {CO}_2$ concentration in the gas phase. Thus, the ${\mathrm {p}}K_{a2}$ value used for the application of Eq. (2) is determined experimentally using the HH Eq. (3) and the initial pH value in the NMR tube, which was measured with a pH meter before the experiment. Averaged over the whole data set, this resulted in a calculated ${\mathrm {p}}K_{a2}$ of $6.15 \pm 0.06$ and $6.02 \pm 0.07$ for the 0.1 M and 1 M experiments, respectively.

Figure 2b,c show the measured pH versus time for $\hbox {KHCO}_3$ concentrations of 0.1 M and 1 M, respectively, and for three representative potentials. A more negative potential results in an accelerated pH increase in both cases. In 0.1 M solution the increase is almost linear, while in 1 M solution a fast increase is observed at the beginning of the experiment, followed by a deceleration of this development. In curves that pass the threshold of pH 7.6 no discontinuity is found, which indicates that the used methods are compatible. For experiments with an initial $\hbox {KHCO}_3$ concentration of 0.1 M, the measured pH was mostly below the threshold pH 7.6. The data range shown in Fig. 2b is therefore larger than in Fig. 2c, where primarily the CCS function was used for pH determination. Increased fluctuations are explained by a larger impact of bubble formation on signal integrals compared to the effect on chemical shifts. For the highest applied potential, the highest rate of bubble formation is observed, which results in increased uncertainties in the pH value for both electrolyte concentrations. The error propagation in the HH (Eq. 3) and CCS method (Eq. 5) is discussed in Sect. S2. The impact of the fluctuations in CCS and the integral of $\hbox {CO}_2$ and $\hbox {HCO}_3^-$ on the error propagation is presented in Sect. S3. It was found that the standard error of the pH value is mainly influenced by errors in the determination of the ${\mathrm {p}}K_{a1}$ and ${\mathrm {p}}K_{a2}$ values. Only when applying the CCS function in the limiting case near ${\mathrm {pH}} = 7.6$, values of the pH standard error are up to 0.15 for 0.1 M solution and 0.29 for 1 M solution. These values are on the same order of magnitude as depicted in Fig. 2b,c.

The evolution of the pH value is in accordance with pH values measured before and after electrolysis using a pH meter, cf. Fig. S7. All curves start at the expected pH value for $\hbox {CO}_2$-saturated $\hbox {KHCO}_3$ solution, i.e. at pH 6.7 for 0.1 M and at pH 7.6 for 1 M. Final pH values at the end of the electrolysis experiment are larger than the values measured with the pH meter. This could be a manifestation of non-equilibrium between carbon species in solution and $\hbox {CO}_2$ in the atmosphere caused by electrolysis, or it could be a local pH effect. Since the liquid–gas interface in the NMR tube is small and the distance between the surface and the WE is sizeable, $\hbox {CO}_2(g) \leftrightarrow \hbox {CO}_2(aq)$ exchange as well as $\hbox {CO}_2$ diffusion may not be fast enough to keep the electrolyte at the WE, which is in the region of the sample that is measured by NMR, in a quasi-equilibrium. After operation, the concentration equilibrates between gas and liquid phase as well as between bulk and WE proximity. Therefore, the pH measured by the pH meter after electrolysis is lower than the final pH measured by *in operando* NMR. The NMR measurements represent a statistical average over the whole NMR sensitive volume around the electrode, which means that even higher pH near the electrode surface could be expected. Since the line width of the carbonate peak is small, no substantial spatial distribution of pH is indicated. Therefore, the equilibration of the pH value inside the sensitive NMR volume is faster than the change of the pH. Such an equilibration would not be expected on the timescale of the experiment if only self-diffusion in aqueous media would be considered. It may be facilitated by diffusion caused by a concentration gradient due to electrolysis at the electrode, or by convection due to Joule heating. Only minor temperature differences can cause sufficient motion in the sample to achieve such an averaging at the employed low current densities. At the same time, as postulated by Varela et al., the local pH of a dilute $\hbox {KHCO}_3$ solution might even exhibit the local pH of a more concentrated solution^28^. The data in Fig. 2b,c show larger difference between measured and initial pH for the more dilute solution, thus confirming such a hypothesis.

**Figure 3:**
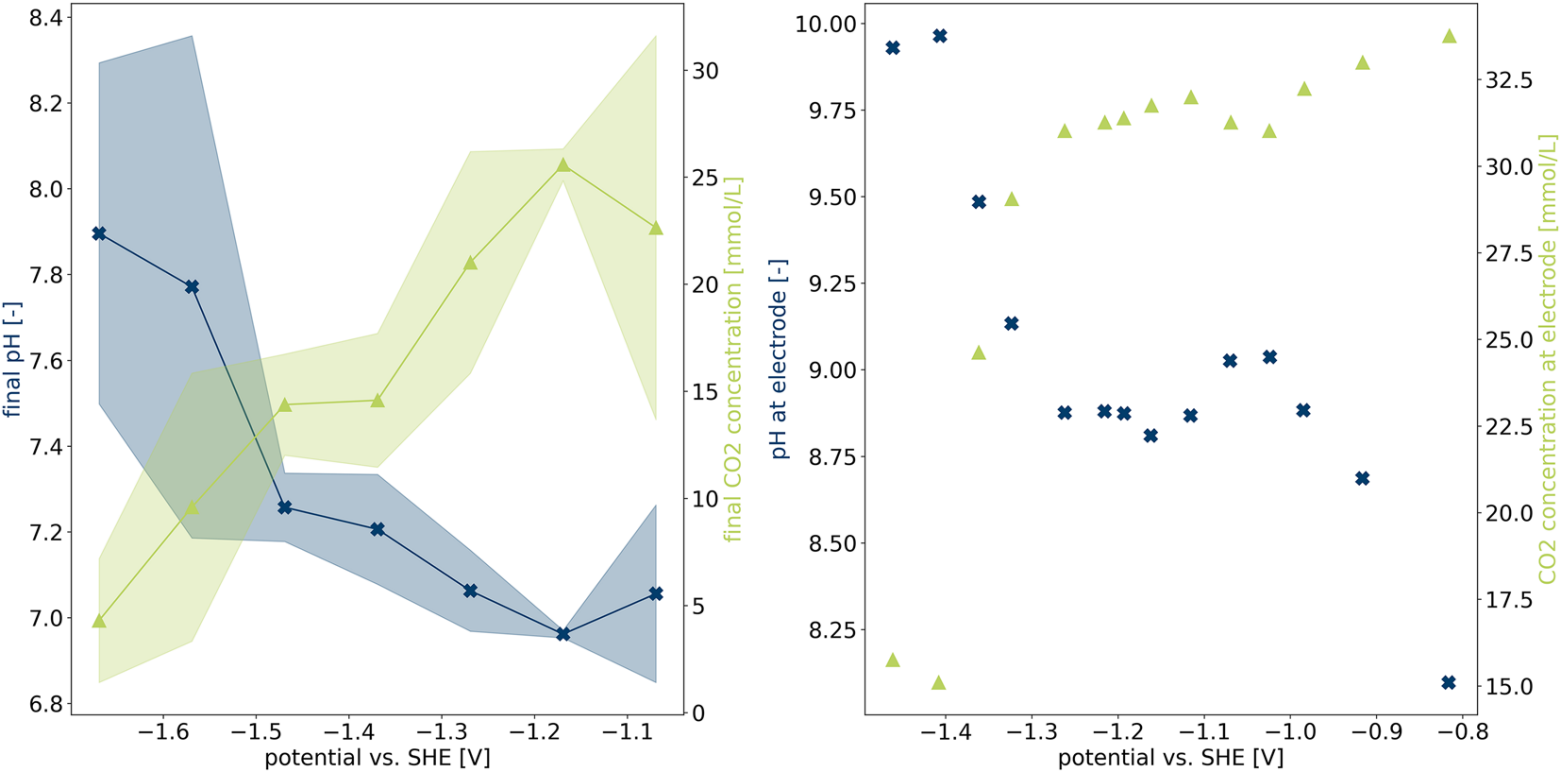
Comparison of experimental and theoretical potential-dependent pH and $\hbox {CO}_2$ concentration. (**a**) pH ($\mathbf {\times }$) and $\hbox {CO}_2$ concentration ($\bigtriangleup$) values determined by $^{13}\hbox {C}$ NMR at the end of a potential step, recorded at potentials between $-1.07$ and $-1.67\,{\mathrm {V}}$. (**b**) Calculated pH (${\times }$) and $\hbox {CO}_2$ concentration ($\bigtriangleup$) at the electrode. Values are taken from Fig. 8 in Gupta et al.^9^. Since final values of the measured average over a volume surrounding the electrode from a batch experiment are compared with steady state values in direct electrode proximity from a computer calculation, this depiction serves as a qualitative comparison only.

The potential-dependent local pH can be qualitatively compared to the calculations by Gupta et al.^9^. Figure 3a depicts the final pH value determined by *in operando* NMR as a measure for the pH in the vicinity of the electrode as well as the final $\hbox {CO}_2$ concentration $[{\hbox {CO}_2}]_{\mathrm {final}}$. It was determined using

6 $$\begin{aligned} {[}{\hbox {CO}_2}]_{\mathrm {final}} = \frac{S_{\mathrm{{CO}}_2,\mathrm{{final}}}}{S_{\mathrm{{CO}}_2,\mathrm{{initial}}}} \times [{\hbox {CO}_2}]_{\mathrm {sat}}, \end{aligned}$$

where an initial saturation concentration $[{\hbox {CO}_2}]_{\mathrm {sat}}$ of 52.7 mM at $10\,^{\circ }\hbox {C}$ is assumed^25^, and $S_{{\mathrm {CO}}_2,{\mathrm {final}}}$ and $S_{{\mathrm {CO}}_2,{\mathrm {initial}}}$ are the final and initial integrals of the $\hbox {CO}_2$ resonance, respectively. Final pH values and $\hbox {CO}_2$ concentrations show an opposing course, i.e. low pH and high $\hbox {CO}_2$ concentration for low potentials and vice versa for high potentials. A plateau between $-1.1$ and $-1.4\,{\mathrm {V}}$ is followed by an abrupt increase in pH and therefore decrease in $\hbox {CO}_2$ concentration at more negative potential. This course can also be observed in the data of Gupta et al., shifted by approximately 100 mV to higher potentials, as depicted in Fig. 3b. Such a shift may be caused by the absence of an iR drop in the theoretical study. Katsounaras et al.^29^ attribute this plateau to inhibition of the HER in the specific local pH region resulting from the applied potentials.

**Figure 4:**
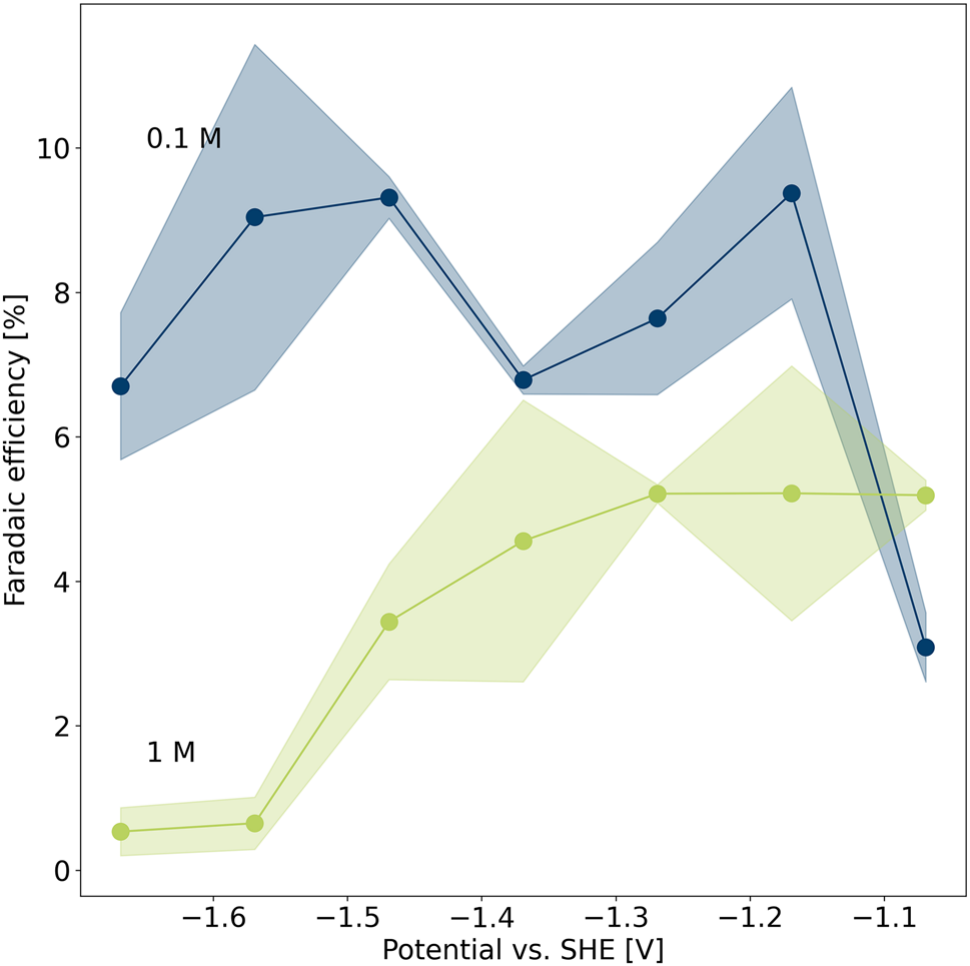
Potential-dependent Faradaic efficiencies of formate production in 1 M (green) and 0.1 M (blue) $\hbox {KHCO}_3$ solution.

After electrolysis, the electrodes are removed from the electrolyte. The liquid solution is then analysed ex situ to detect liquid products that have formed during electrolysis. Reference compound trimethylsilylpropanoic acid (TSP) is added to obtain a concentration of 1 mM and the sample is investigated by $^{1}\hbox {H}$ NMR with water suppression at a magnetic field of 18.8 T (800 MHz for $^{1}\hbox {H}$). Formate is the major liquid product in Cu-catalysed $\hbox {CO}_2$RR at the employed current densities^8^. Other $^{13}\hbox {C}$-labelled molecules could not be identified in our experiments, cf. Fig. S8 for the $^{1}\hbox {H}$ spectra. Low selectivity for these products, a polycrystalline and untreated Cu surface, or even contamination by $\hbox {Ag}^+$ ions from the reference electrode could be possible reasons^30^. However, potential-dependent Faradaic efficiencies (FE) of formate, depicted in Fig. 4, show varying selectivity for this reaction pathway for the initial carbonate concentrations in consideration. The reaction pathway to formate is reported as a “dead-end road”, as it is not further reduced on a Cu electrode^3^, although others suggest a possible pathway to methanol^31^. However, it competes with the pathway to CO and its further reduction products, i.e. methane, ethylene and other short-chain hydrocarbons. Therefore, the formation of formate is used to distinguish the favoured reaction pathway under varying conditions^32^. Since anode and cathode are not separated by a membrane in the electrochemical cell, it also cannot be ruled out that products formed at the WE are subsequently oxidised at the CE. This will not influence the local pH measurements at the WE, but may explain why the measured values are lower compared to literature^8^. In general, FE for formate is lower over the whole potential range for the more concentrated solution. At high potentials, the FE approaches zero. This is expected, as at high potential and/or strongly basic local conditions formate formation was found to be suppressed due to progressing desorption of bicarbonate^16^. Simultaneously, high FE for HER were reported for these conditions^8^. The strong suppression of formate formation at the most negative potentials could also be caused by the dynamic change of surface pH: Fig. 2c shows a rapid increase in local pH in the first minutes of operation, inhibiting any further formate formation during electrolysis.

The suppression of formate formation is less pronounced in 0.1 M solution as the pH value at the beginning of the experiment is lower compared to the 1 M solution. Only towards the end of the experiment the increase in local pH inhibits formate formation. In the investigated potential range, the formate formation in the diluted solution is therefore almost potential-independent.

## Conclusion

*In operando* determination of pH in electrode proximity in a bicarbonate/carbonate electrolyte system using $^{13}\hbox {C}$ NMR spectroscopy is demonstrated. In a pH range between approximately 4–12, a combination of titration curve of the carbonate chemical shift and the Hendersson–Hasselbalch equation has proven to be robust even when the system is disturbed by the formation of gas bubbles. The potential dependent changes of local pH and $\hbox {CO}_2$ concentration are in qualitative accordance with simulated data from literature. The experiments demonstrate a significant increase in local pH, emphasising that the bulk pH cannot be assumed to correctly describe reaction conditions in direct electrode vicinity. Dilute electrolytes with a low bulk pH but also low buffer capacity might even result in higher local pH than found in a concentrated electrolyte. Ex situ product analysis indicates that the formate production is consistent with such a dependence on the local pH, influenced by the applied potential and the buffer capacity of the electrolyte.

This work presents a method that can be easily applied to a wide range of electrode materials and can facilitate the determination of real reaction conditions in terms of local pH and $\hbox {CO}_2$ concentration. It presents a complement to infrared-based methods, which are selective to a thinner surface layer and require optical accessibility. Here the method was demonstrated for a local volume surrounding an electrode that was limited by spatial restriction of the sample geometry. The combination with magnetic resonance imaging to achieve a higher spatial resolution will be presented elsewhere.

## Methods

The *in operando* electrolysis cell and the shielding setup were constructed as described previously^25^. In the following, only changes in the cell setup and the experimental procedure are mentioned.

### Electrochemical cell

**Figure 5:**
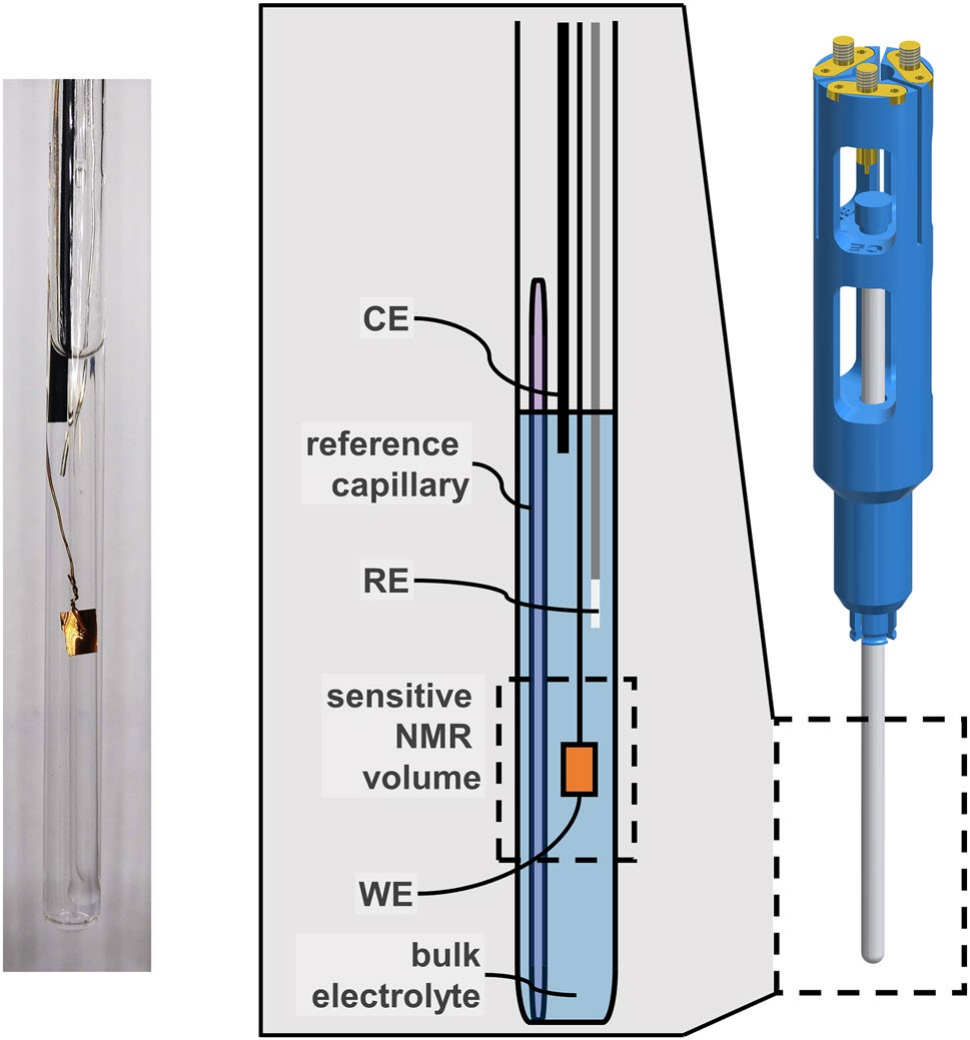
Electrochemical cell for *in operando* $^{13}\hbox {C}$ NMR. (**a**) Photograph and (**b**) schematic of the cell, including copper working electrode (WE), chlorinated silver wire reference electrode (RE), graphite rod counter electrode (CE), and reference capillary. (**c**) Model of the 3D printed sample holder fitting onto the NMR probe.

Copper foil (GoodFellow GmbH, Hamburg, Germany) with outer dimensions of $2.5\,{\mathrm {mm}} \times 4\,{\mathrm {mm}} \times 0.05\,{\mathrm {mm}}$ is perforated and the stripped end of a copper wire (GoodFellow GmbH, Hamburg, Germany) with 0.15 mm diameter and 0.025 mm PTFE insulation is pulled through the hole and twisted around itself to ensure contacting. For every experiment a new working electrode is used. Other electrodes are rinsed with demineralised water and reused. A capillary containing $^{13}\hbox {C}$-labelled acetonitrile (99 atom%, $^{13}\hbox {CH}_3^{12}\hbox {CN}$, Sigma Aldrich, Munich, Germany) was introduced into the cell as an external NMR reference. The liquid was pulled into a $50\,\upmu \hbox {L}$ capillary pipette (Hirschmann Laborgeräte GmbH & Co. KG, Eberstadt, Germany) by capillary forces before melting both ends of the capillary. The closed capillary was placed into the NMR tube in a way that the reference liquid is equally distributed in the sensitive volume of the probe. The reference substance did not only serve as a chemical shift reference, with a $^{13}\hbox {C}$ chemical shift of 4.43 ppm versus TSP at $10\,^{\circ }\hbox {C}$, but it could also be used to correct for amplitude fluctuations and phase drifts that may be caused by variations of the tuning mode during an experiment. Qualitatively, it indicated line shape changes induced by bubbles as well, but since these changes are different for the electrolyte and the reference sample, a correction is challenging and has not been attempted. The temperature-dependent $^{13}\hbox {C}$ chemical shift of $^{13}\hbox {CH}_3^{12}\hbox {CN}$, $\delta _{{{{\mathrm{CH}}_3{\mathrm{CN}}}}}$, referenced to TSP is depicted in Fig. S1. The linear correlation,

7 $$\begin{aligned} \delta _{{\mathrm {CH}}_3{\mathrm {CN}}} = -0.0104\,\frac{{\mathrm {ppm}}}{^{\circ }\hbox {C}} \times T + 4.5308\,{\mathrm {ppm}} \, , \end{aligned}$$

where *T* is the temperature in $^{\circ }\hbox {C}$, is used to estimate temperature gradients in the sample due to radio frequency excitation. A schematic of the electrochemical cell and the full assembly is depicted in Fig. 5.

### Electrolyte preparation

$^{13}\hbox {C}$-enriched stock solutions of $\hbox {KHCO}_3$ (98 atom %, Sigma Aldrich Chemie GmbH, Munich, Germany) were prepared at 0.1 M and 1 M concentrations and pre-electrolysed as described by Hori et al.^33^ in order to remove impurities such as heavy metal ions, which will otherwise negatively affect the performance of the electrolysis. 20 mL of the respective stock solution were filled into a twin-necked flask connected to a Schlenk line and degassed by three freeze–pump–thaw cycles. Using two $5\,{\mathrm {mm}} \times 5\,{\mathrm {mm}}$ platinum mesh electrodes (GoodFellow GmbH, Hamburg, Germany) a constant current of $150\,\upmu \hbox {A}$ was applied for 20 h while stirring with *ca.* 250 rpm using a magnetic stirrer. $600\,\upmu \hbox {L}$ of stock solution were filled in a NMR tube and bubbled with $^{13}\hbox {C}$-enriched $\hbox {CO}_2$ (99 atom %, Sigma Aldrich, Munich, Germany) until saturation for *ca.* 20 min at a flow rate of *ca.* 0.3 ${\mathrm {mL\,s}}^{-1}$. To maintain temperature stability, the sample was kept in a water bath at $10\,^{\circ }\hbox {C}$ during bubbling. The pH value was measured after $\hbox {CO}_2$ saturation and later after electrolysis using a benchtop Mettler–Toledo FiveEasy pH meter with a Mettler–Toledo InLab NMR pH electrode (Mettler–Toledo GmbH, Giessen, Germany) for direct pH measurement of the sample in the NMR tube. The three-electrode setup including the reference capillary was introduced afterwards into the NMR tube and the tube sealed with a gas tight cap. The NMR tube was mounted using a 3D-printed holder that fits onto the NMR probe and provides connectors for the electrodes to the potentiostat (Fig. 5c). The probe was pre-tempered at $10\,^{\circ }\hbox {C}$ to avoid degassing due to transient temperature effects during thermal equilibration.

### Electrochemical parameters

The electrodes of the electrochemical cell were connected to a BioLogic SP-200 potentiostat (BioLogic Science Instruments, Seyssinet-Pariset, France). The employed micro reference electrode exhibited a steady open circuit voltage (OCV) of $0.115 \pm 0.002\,{\mathrm {V}}$ versus a commercial Ag/AgCl (3 M KCl) reference electrode (Deutsche METROHM GmbH & Co. KG, Filderstadt, Germany) in a 1 M $\hbox {KHCO}_3$ solution. Potentials $E_{\hbox {microAg/AgCl}}$ measured with the micro reference electrode are converted to the standard hydrogen electrode (SHE) scale according to

8 $$\begin{aligned} E_{\mathrm {SHE}} = E_{\hbox {microAg/AgCl}} + 0.205\,{\mathrm {V}} - 0.73\times 10^{-3}\,{\mathrm {V}}/^{\circ }{\mathrm {C}} \times (T-25\,^{\circ } {\mathrm {C}}) + 0.115\,{\mathrm {V}} \, , \end{aligned}$$

where *T* is the temperature in $^{\circ }\hbox {C}$^34^. Chronoamperometric (CA) measurements were conducted for 2 h at constant potential, with seven data points in the range of [${\mathrm {-1.67\,V}}$,${\mathrm {-1.07\,V}}$] versus SHE and two repetitions. Results of CA measurements of 0.1 M and 1 M solutions are depicted in Fig. S2. Figure S2a,b show that bubble formation has an effect on the evolution of current density as well, which exhibits increasing fluctuations with more negative potential.

### NMR parameters

The cell was inserted into a Bruker DiffBB broadband gradient probe on a Bruker Avance III HD spectrometer (Bruker BioSpin GmbH, Rheinstetten, Germany) with a 9.4 T wide-bore magnet, corresponding to a $^{13}\hbox {C}$ resonance frequency of 100.6 MHz. The cell holder presented in our previous publication^25^ was adjusted such that the cell can be inserted from the top of the magnet with a narrow-bore sample lift installed. During CA, $^{13}\hbox {C}$ spectra were continuously recorded using $30^{\circ }$-pulses with $^{1}\hbox {H}$ decoupling (WALTZ-16 sequence with 128 repetitions every 2 s), resulting in acquisition of one spectrum every 6 min. A pulse length of $4\,\upmu \hbox {s}$ and a radio frequency power of 40.996 W was set. Spectra were processed with 1 Hz line broadening and zero-filling.

After the *in operando* experiment, electrodes and reference capillary were removed from the NMR tube and the sample was analysed ex situ to study the formation of liquid products. To enable quantification, a well-defined amount of the reference substance TSP was added to the sample, which resulted in a concentration of 1 mM. For higher sensitivity, $^{1}\hbox {H}$ NMR with water suppression using excitation sculpting with perfect echo was performed^35^. In addition, the sample was analysed at higher magnetic field of 18.8 T (800 MHz for $^{1}\hbox {H}$) using a Bruker DiffBB probe. The relaxation delay was set to 5 s, and 256 scans were acquired. The pulse length was $21\,\upmu \hbox {s}$ and the pulse power was 27.58 W. The pulse length of the sine-shaped selective pulse was $800\,rmu\hbox {s}$ and the respective pulse power was 0.21943 W. Resulting $^{1}\hbox {H}$ spectra are depicted in Fig. S8, showing the formate resonance doublet at 8.46 ppm. The Faradaic efficiency (FE) of formate was calculated according to

9 $$\begin{aligned} {\mathrm {FE}} = \frac{S_{\mathrm {formate}}}{S_{\mathrm {TSP}}} \times 9 \times 2 \times 1\,{\mathrm {mmol/L}} \times 600\,\upmu {\mathrm {L}} \times \frac{F}{\int _{0}^{2\,{\mathrm {h}}} I_{\mathrm {CA}}(t) \,{\mathrm {d}}t}, \end{aligned}$$

where $S_{\mathrm {formate}}$ and $S_{\mathrm {TSP}}$ are the integrals of the formate and TSP resonances, respectively, *F* is the Faraday constant and $I_{\mathrm {{CA}}}(t)$ is the current as a function of time during the CA experiment. The factor 9 in Eq. (9) results from the ratio of equivalent protons in TSP and formate, and the factor 2 represents the number of electrons transferred per formate molecule formed.

## Supplementary Information

Supplementary Information.

## Data Availability

All data reported in this work are available from the corresponding author M.S. by request.
