# Supplementary material for: Quantifying local pH changes in carbonate electrolyte during copper-catalysed CO2 electroreduction using in operando 13C NMR
Source: Sci Rep. 2022 May 18;12:8274. doi: 10.1038/s41598-022-12264-8 (PMC9117298; doi:10.1038/s41598-022-12264-8)
Supplement: Supplementary file 1 — Supplementary Information. [file 41598_2022_12264_MOESM1_ESM.pdf]

Supporting Information for

Quantifying Local pH Changes in Carbonate Electrolyte during Copper-Catalysed CO_2 Electroreduction Using *In Operando* ^{13}C NMR

Michael Schatz[1,2,*], Sven Jovanovic[1], Rüdiger-A. Eichel[1,3], and Josef Granwehr[1,2]

[1]Institute of Energy and Climate Research, Fundamental Electrochemistry (IEK-9), Forschungszentrum Jülich, Jülich, 52425, Germany
[2]Institute of Technical and Macromolecular Chemistry, RWTH Aachen University, Aachen, 52074, Germany
[3]Institute of Physical Chemistry, RWTH Aachen University, Aachen, 52074, Germany
[*]m.schatz@fz-juelich.de

S1 pH dependent carbonate chemical shift

The Hendersson–Hasselbalch (HH) equation can be applied to the equilibrium between the carbonate species ${HCO_3}^-$ and ${CO_3}^{2-}$,

$$\mathrm{pH} = \mathrm{pK_{a1}} + \log_{10}\frac{[{CO_3}^{2-}]}{[{HCO_3}^-]} \tag{1}$$

$$\Leftrightarrow \frac{[{HCO_3}^-]}{[{CO_3}^{2-}]} = 10^{\mathrm{pK_{a1}}-\mathrm{pH}}\,. \tag{2}$$

The measured chemical shift δ_c of the carbonate peak can be expressed as a function of the concentrations of the carbonate species,

$$\left|\frac{\delta_c - \delta_{{HCO_3}^-}}{\delta_{{CO_3}^{2-}} - \delta_{{HCO_3}^-}}\right| = \frac{[{CO_3}^{2-}]}{[{CO_3}^{2-}] + [{HCO_3}^-]} = \frac{1}{1+\frac{[{HCO_3}^-]}{[{CO_3}^{2-}]}}\,. \tag{3}$$

Inserting equation 2 in equation 3 results in the function that is fitted to experimental data,

$$\delta_c = \delta_{{HCO_3}^-} + \frac{\delta_{{CO_3}^{2-}} - \delta_{{HCO_3}^-}}{1+10^{\mathrm{pK_{a1}}-\mathrm{pH}}}\,, \tag{4}$$

where the $\delta_{{HCO_3}^-}$ is the chemical shift of ${HCO_3}^-$ and $\delta_{{CO_3}^{2-}}$ is the chemical shift of ${CO_3}^{2-}$. Both are predetermined by fitting of a titration curve.

S2 Propagation of uncertainty

An estimate for the error of the described pH quantification method can be obtained using a simplified error propagation discussion that assumes uncorrelated errors for the different experimental parameters. Both the carbonate chemical shift (CCS) method as well as the HH method share the same functional form,

$$\mathrm{pH} = \mathrm{p}K_a + \log\frac{\alpha}{\beta}\,, \tag{5}$$

where α represents $[HCO_3^-]$ for the HH and $(\delta_c - \delta_{HCO_3^-})$ for CCS method, and β represents $[CO_2]$ for the HH and $(\delta_{CO_3^{2-}} - \delta_c)$ for the CCS method. The standard error of the pH, s_{pH}, can be estimated as

$$s_{pH} = \sqrt{\left(\frac{\partial pH}{\partial pK_a}\right)^2 s_{pK_a}^2 + \left(\frac{\partial pH}{\partial \alpha}\right)^2 s_\alpha^2 + \left(\frac{\partial pH}{\partial \beta}\right)^2 s_\beta^2}$$

$$= \sqrt{s_{pK_a}^2 + \left(\frac{s_\alpha}{\ln(10)\alpha}\right)^2 + \left(\frac{s_\beta}{\ln(10)\beta}\right)^2}, \tag{6}$$

where s_{pK_a}, s_α and s_β represent the standard errors of pK_a, α and β, respectively. In other words, the standard error of the pH value combines the standard error of pK_a with the relative errors of α and β. Even though logarithmic functions with an argument far away from 1 are not particularly well suited for a linear approximation as used by Equation 12, the result is qualitatively plausible. Any pH detection method that relies on the determination of a ratio of protonated and deprotonated species becomes inaccurate once only one species is present in detectable quantities.

Using Equation 12, the standard error for the HH method can be written as

$$s_{pH} = \sqrt{s_{pK_{a2}}^2 + 0.189\left(\frac{s_{[HCO_3^-]}}{[HCO_3^-]}\right)^2 + 0.189\left(\frac{s_{[CO_2]}}{[CO_2(aq)]}\right)^2}, \tag{7}$$

where $s_{[CO_2]}$ and $s_{[HCO_3^-]}$ are the standard error of $[CO_2]$ and $[HCO_3^-]$, respectively. For the CCS method,

$$s_{pH} = \sqrt{s_{pK_{a1}}^2 + 0.189\left(\frac{s_{\delta_c - \delta_{HCO_3^-}}}{\delta_c - \delta_{HCO_3^-}}\right)^2 + 0.189\left(\frac{s_{\delta_{CO_3^{2-}} - \delta_c}}{\delta_{CO_3^{2-}} - \delta_c}\right)^2}, \tag{8}$$

where $s_{\delta_c - \delta_{HCO_3^-}}$ and $s_{\delta_{CO_3^{2-}} - \delta_c}$ are the standard error of $\delta_c - \delta_{HCO_3^-}$ and $\delta_{CO_3^{2-}} - \delta_c$, respectively. Alternatively, equation 8 can be rewritten in terms of the individual standard errors s_{δ_c}, $s_{\delta_{HCO_3^-}}$ and $s_{\delta_{CO_3^{2-}}}$ of δ_c, $\delta_{HCO_3^-}$ and $\delta_{CO_3^{2-}}$, respectively, as

$$s_{pH} = \sqrt{s_{pK_{a1}}^2 + 0.189\left(\frac{s_{\delta_c}}{\delta_c - \delta_{HCO_3^-}} + \frac{s_{\delta_c}}{\delta_{CO_3^{2-}} - \delta_c}\right)^2 + 0.189\left(\frac{s_{\delta_{HCO_3^-}}}{\delta_{HCO_3^-} - \delta_c}\right)^2 + 0.189\left(\frac{s_{\delta_{CO_3^{2-}}}}{\delta_c - \delta_{CO_3^{2-}}}\right)^2}. \tag{9}$$

Here the partial derivatives

$$\frac{\partial pH}{\partial \delta_c} = \left[\frac{1}{\delta_c - \delta_{HCO_3^-}} + \frac{1}{\delta_{CO_3^{2-}} - \delta_c}\right] \Big/ \ln(10) \tag{10}$$

$$\frac{\partial pH}{\partial \delta_{HCO_3^-}} = \frac{1}{\ln(10)(\delta_{HCO_3^-} - \delta_c)} \tag{11}$$

$$\frac{\partial pH}{\partial \delta_{CO_3^{2-}}} = \frac{1}{\ln(10)(\delta_c - \delta_{HCO_3^-})} \tag{12}$$

have been used.

It is apparent from the equations for the standard error of a pH value that at the fringes of the validity range of a particular technique the error starts to diverge. The farther the pH from a pK_a value, the larger s_{pH} becomes, even if the error of a measured chemical shift or concentration value remains constant. This is particularly relevant if bubbles increase the uncertainty of the measured values relative to the error of the pK_a.

Since the pK_a value used for data analysis is typically only determined once for a particular set of measurements, its error appears as a systematic error. If it cannot be neglected compared to the other errors, it needs to be added manually to the error obtained from experiment repetitions.

S3 Error estimation

To estimate the errors caused by bubble formation at the electrode, the fluctuation of the fitted peak properties, *i.e.* integral, chemical shift and Full Width Half Maximum (FWHM), are determined in terms of standard deviation of the measured values

	0.1 M	1 M
$s_{\mathrm{p}K_{a2}}$	0.06	0.07
$\frac{s_{[HCO_3^-]}}{[HCO_3^-]}$	0.0344	0.0379
$\frac{s_{[CO_2]}}{[CO_2(aq)]}$	0.0663	0.0901
s_{pH}	0.0682	0.0819

Table 1. Error values for the determination of s_{pH} according to the error propagation described in equation 7.

	0.1 M		1 M	
	$\delta_c = \mathrm{p}K_{a1}$	$\delta_c = 7.6$	$\delta_c = \mathrm{p}K_{a1}$	$\delta_c = 7.6$
$s_{\mathrm{p}K_{a2}}$	0.011		0.011	
s_{δ_c}	0.0137		0.0262	
$\delta_c - \delta_{HCO_3^-}$	3.874	0.039	3.874	0.039
$CO_3^{2-} - \delta_c$	3.871	7.705	3.871	7.705
s_{pH}	0.011	0.154	0.011	0.291

Table 2. Error values for the determination of s_{pH} according to the error propagation described in equation 9.

from a 5-step moving average. The resulting errors are plotted in Figure S6. The integral fluctuations are referenced to their initial value. To assess the uncertainties in the measurement of the carbonate chemical shift (CCS), the fluctuations of chemical shift and FWHM of carbonate and reference peak are referenced to the total change of CCS during the experiment. High error values of the CO_2 peak can be attributed to the depletion of CO_2 during the experiment and therefore poor signal-to-noise ratio.

To quantify the maximum errors resulting from the error propagation due to bubble formation, the presented fluctuation values are used as standard errors in equation 7 and 9. For the estimation of the standard error of the pH value calculated using the HH equation, the values in table 1 are inserted into equation 7. The errors of $\mathrm{p}K_{a2}$ result from statistical averaging. For the determination of s_{pH} of the CCS method, it is assumed that the terms in equation 9 including $s_{\delta_{HCO_3^-}}$ and $s_{\delta_{CO_3^{2-}}}$ can be neglected. These errors originate exclusively from fitting of the titration function of equation 4 to experimental data, which resulted in a coefficient of determination of $R^2 = 0.999$. The remaining variables to be inserted into equation 9 are noted in table 2. Both limiting cases for δ_c are considered, *i.e.* $\delta_c = \mathrm{p}K_{a1} = 9.645$ and $\delta_c = 7.6$. The standard errors for peak integral and chemical shift in table 1 and 2 are values averaged over the whole data set.

Supporting Figures

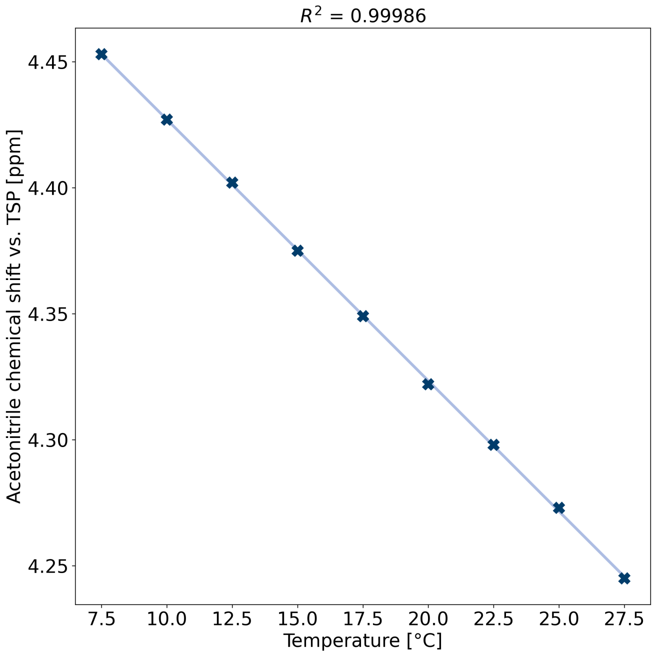

Figure S1. Temperature-dependent chemical shift of acetonitrile reference vs. trimethylsilylpropanoic acid (TSP)

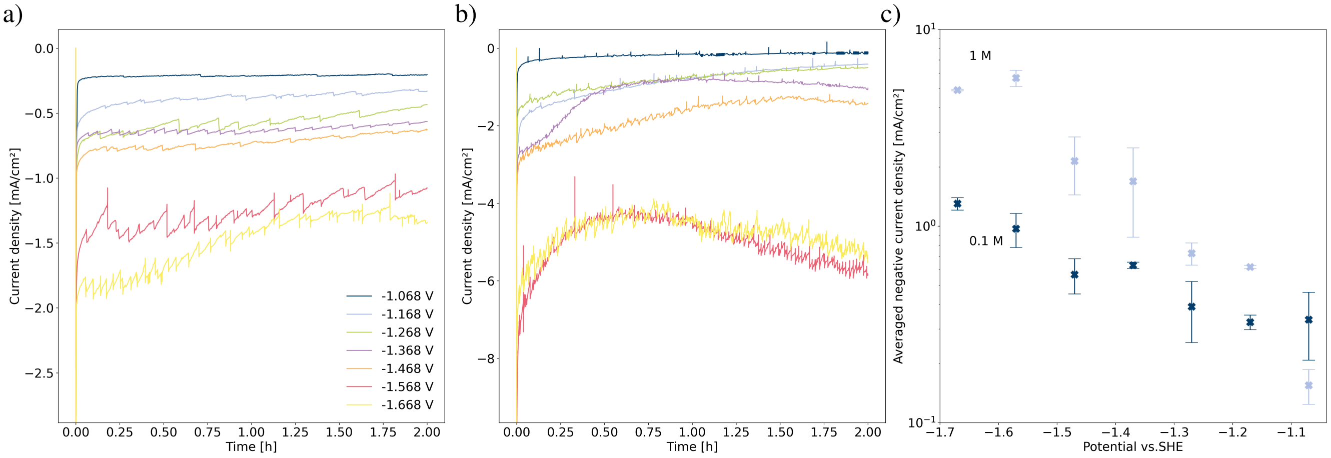

Figure S2. Results of CA measurements. a)-b) Evolution of the current density for 0.1 M and 1 M $KHCO_3$ solution, respectively; c) Averaged negative current density during experiments with varying applied potential.

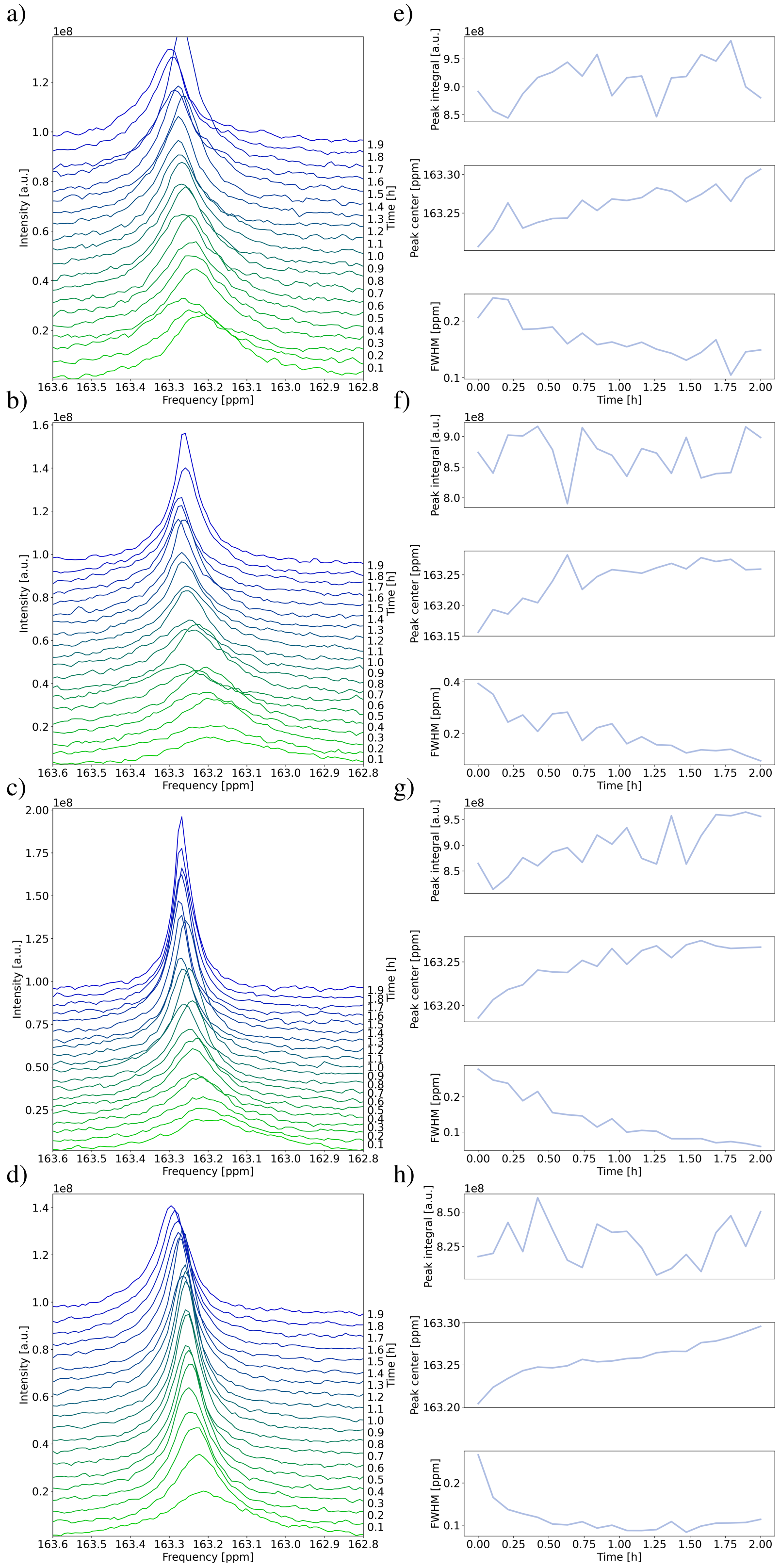

Figure S3. Results of *in operando* ^{13}C NMR measurements with 0.1 M initial $KHCO_3$ concentration. a)-d) Waterfall plots showing the change of the carbonate resonance during 2 h of electrolysis at −1.07 V, −1.27 V, −1.47 V and −1.67 V, respectively; e)-h) Evolution of the carbonate peak integral, chemical shift and FWHM during 2 h of electrolysis at −1.07 V, −1.27 V, −1.47 V and −1.67 V, respectively.

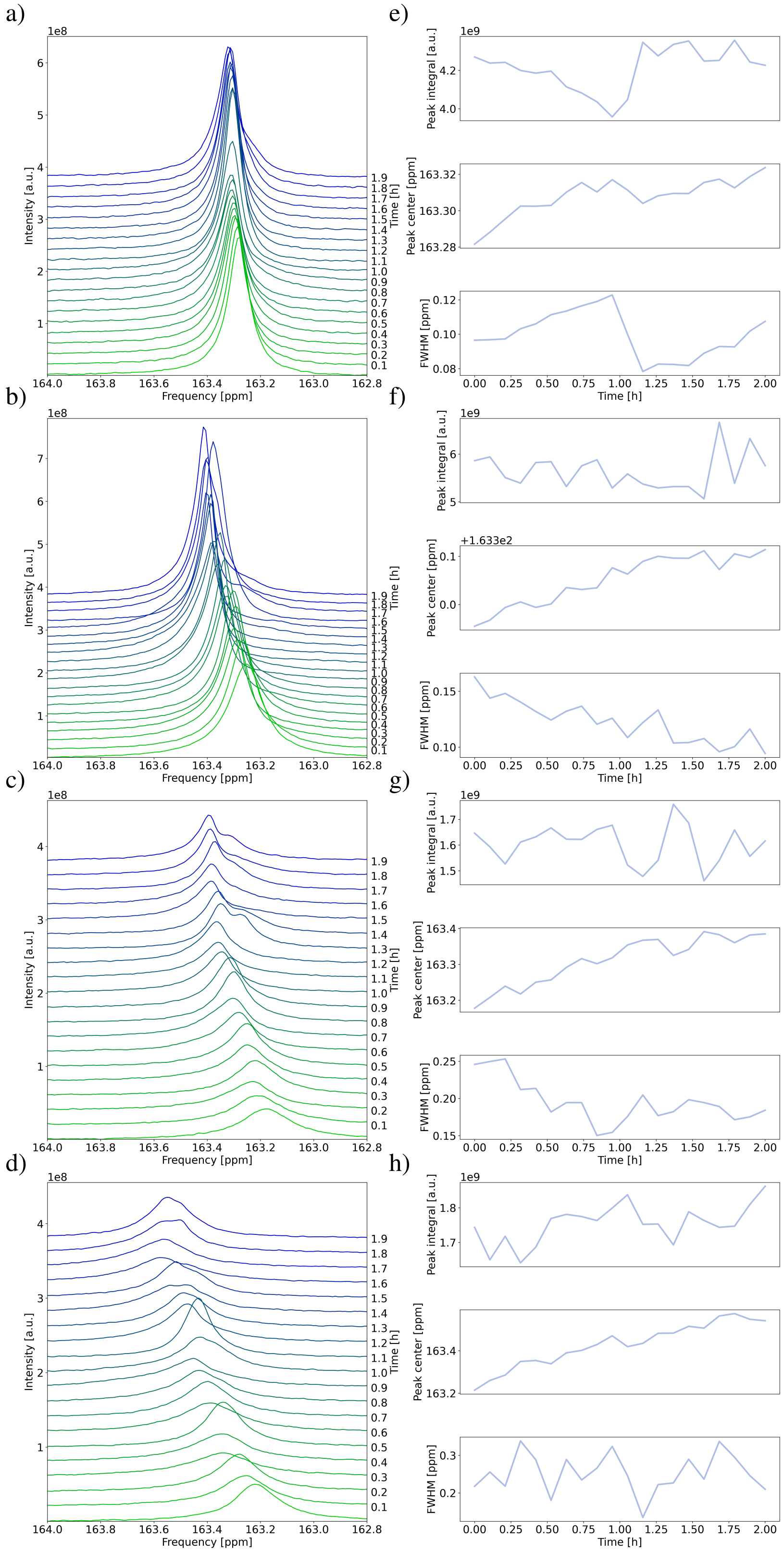

Figure S4. Results of *in operando* ^{13}C NMR measurements with 1 M initial $KHCO_3$ concentration. a)-d) Waterfall plots showing the change of the carbonate resonance during 2 h of electrolysis at −1.07 V, −1.27 V, −1.47 V and −1.67 V, respectively; e)-h) Evolution of the carbonate peak integral, chemical shift and FWHM during 2 h of electrolysis at −1.07 V, −1.27 V, −1.47 V and −1.67 V, respectively.

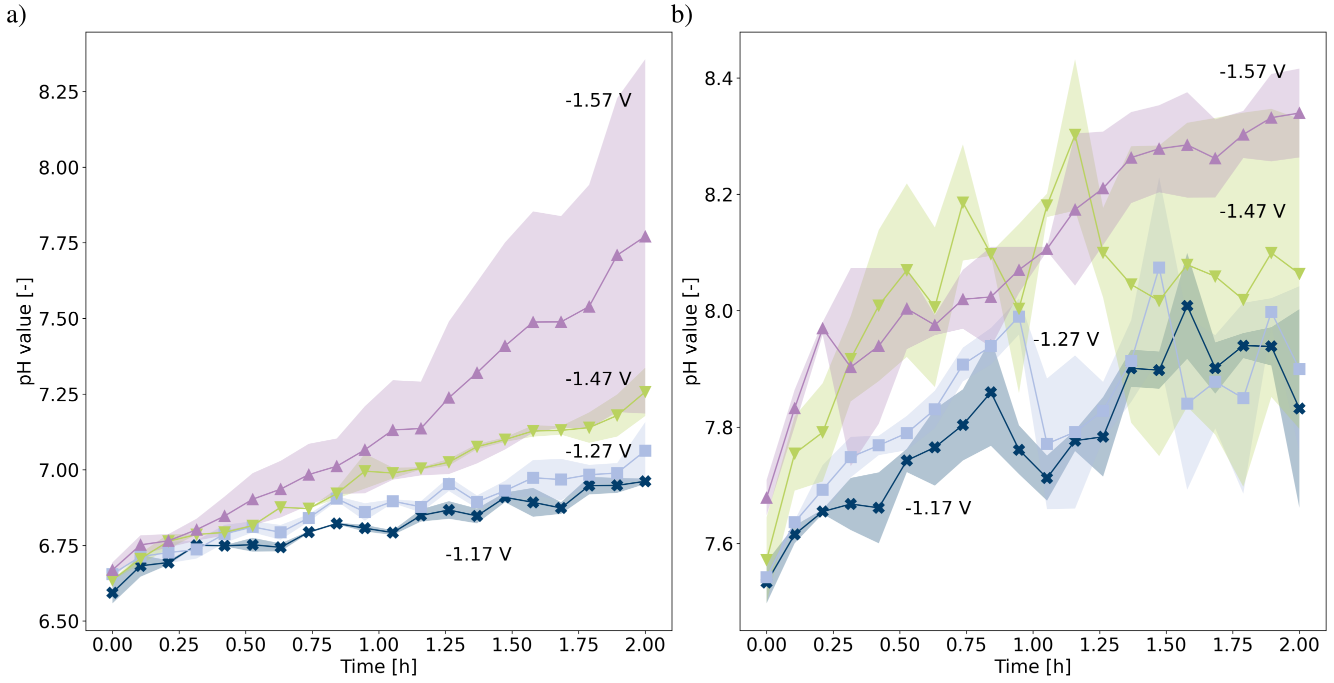

Figure S5. a)-b) pH values as a function of time for initial electrolyte concentration of 0.1 M and 1 M, respectively.

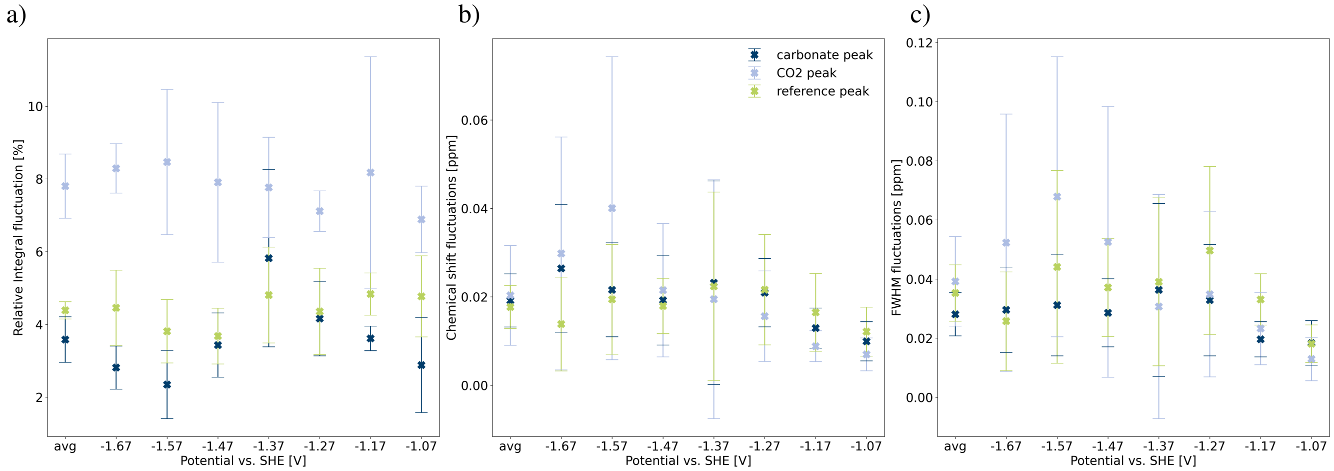

Figure S6. Estimation of errors due to bubble formation. a)-c) Fluctuations of carbonate, CO_2 and reference peak in terms of integral, chemical shift and FWHM, respectively. Fluctuation values are determined as standard deviation between measured values and a 5-step moving average. The fluctuations in the integral values are given relative to their initial value.

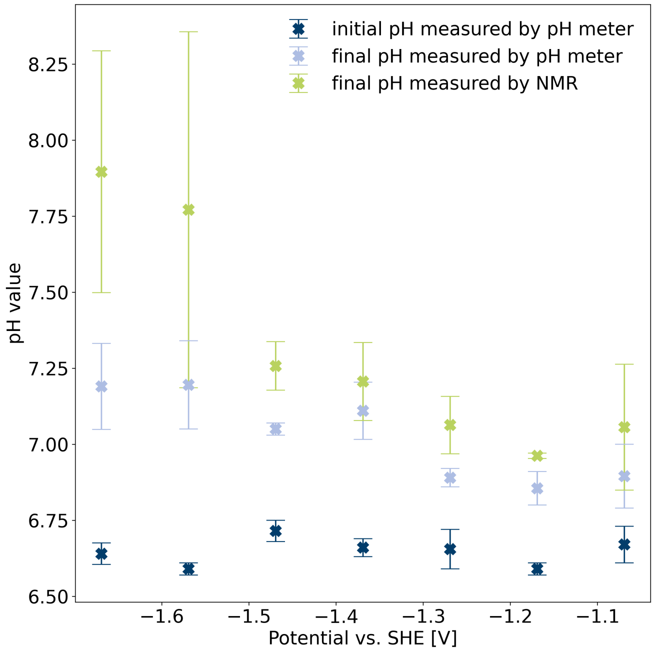

Figure S7. pH values measured by pH meter in the NMR tube before and after the electrolysis operation using 0.1 M initial $KHCO_3$ concentration in comparison with final values determined by *in operando* NMR.

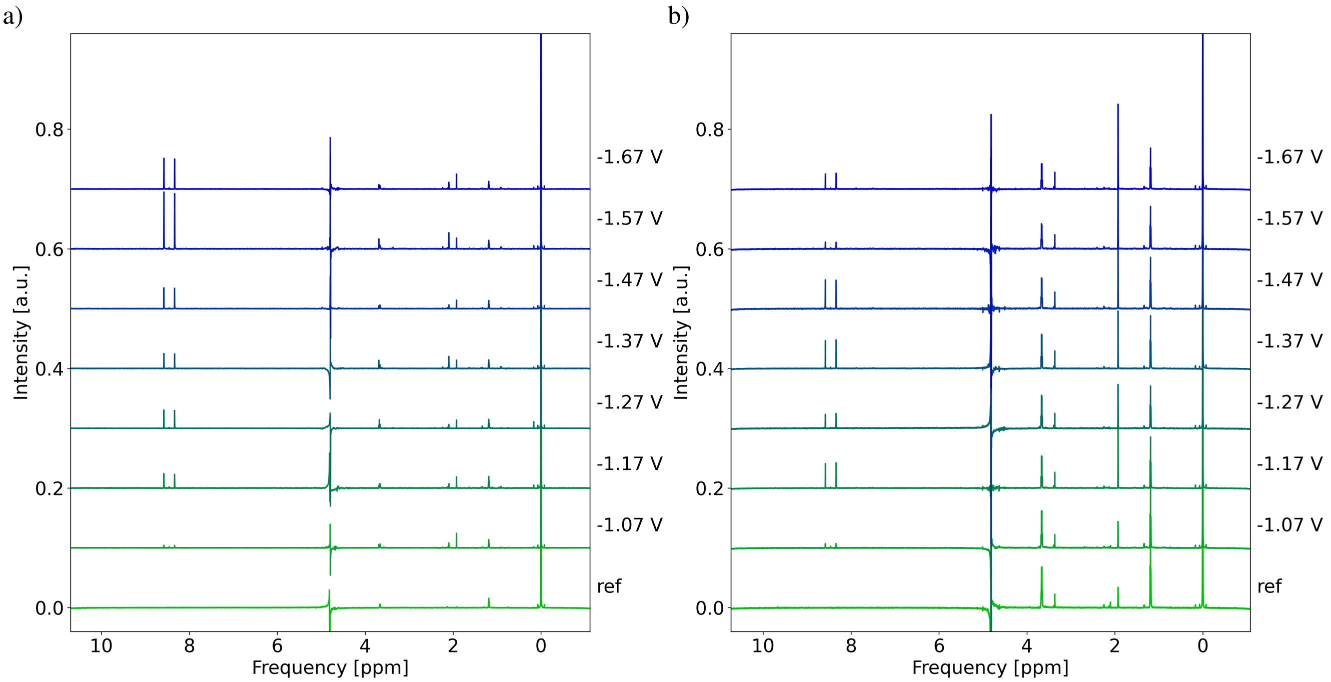

Figure S8. a)-b) ^{1}H *ex situ* NMR spectra of the electrolyte solution after electrolysis with varying potential for initial electrolyte concentration of 0.1 M and 1 M, respectively. Spectra marked with "ref" represent measurements of a sample of the respective stock solution without any potential applied to distinguish between impurities and reaction products.
